# Transcellular migration of neutrophil granulocytes through the blood-cerebrospinal fluid barrier after infection with *Streptococcus suis*

**DOI:** 10.1186/1742-2094-8-51

**Published:** 2011-05-18

**Authors:** Corinna Wewer, Annette Seibt, Hartwig Wolburg, Lilo Greune, M Alexander Schmidt, Jürgen Berger, Hans-Joachim Galla, Ulrike Quitsch, Christian Schwerk, Horst Schroten, Tobias Tenenbaum

**Affiliations:** 1 Department of General Pediatrics, University Children's Hospital, Heinrich-Heine-University, Düsseldorf, Germany; 2 Institute for Genetics of Heart Diseases, Department of Cardiology and Angiology, University Hospital Münster, Germany; 3 Institute of Pathology, University of Tuebingen, Tuebingen, Germany; 4 Institute of Infectiology, Centre for Molecular Biology of Inflammation (ZMBE), Westfälische Wilhelms-University, Münster, Germany; 5 Max Planck Institute of Developmental Biology, Tuebingen, Germany; 6 Department of Biochemistry, Westfälische Wilhelms-University, Münster, Germany; 7 Pediatric Infectious Diseases, Department of Pediatrics, University Hospital Mannheim, Heidelberg University, Mannheim, Germany

## Abstract

**Background:**

A critical point during the course of bacterial meningitis is the excessive influx of polymorphnuclear neutrophils (PMNs) from the blood into the brain. Both paracellular and transcellular routes of leukocyte transmigration through the blood-brain barrier have been described in CNS diseases so far. Thus, we investigated the mechanism of PMN transmigration through the blood-CSF barrier under inflammatory conditions.

**Methods:**

In an "inverted" Transwell culture model of the blood-CSF barrier, the zoonotic agent *Streptococcus suis *(*S. suis*) was used to stimulate porcine choroid plexus epithelial cells (PCPECs) specifically from the physiologically relevant basolateral side. Barrier function was analyzed by measuring TEER and TR-dextran-flux, and tight junction morphology was investigated by immunofluorescence. Route and mechanism of PMN transmigration were determined by immunofluorescence, electron microscopy and FACS analysis. Quantitative real time-PCR was used to determine expression levels of ICAM-1 and VCAM-1.

**Results:**

Here, we show that the transmigration of PMNs through PCPECs was significantly higher after stimulation with TNFα or infection with *S. suis *strain 10 compared to its non-encapsulated mutant. Barrier function was not significantly affected by PMN migration alone, but in combination with *S. suis *infection. Tight junction and cytoskeletal actin reorganisation were also observed after stimulation with *S. suis *or TNFα. Most strikingly, PMNs preferentially migrated across PCPECs via the transcellular route. Extensive sequential analyses of the PMN transmigration process with Apotome^®^-imaging and electron microscopy revealed that paracellular migrating PMNs stop just before tight junctions. Interestingly, PMNs subsequently appeared to proceed by transcellular migration via funnel-like structures developing from the apical membrane. It is noteworthy that some PMNs contained bacteria during the transmigration process. Flow cytometric and transmigration inhibition studies with integrin-specific antibodies showed that PMN traversal is dependent on CD11b/CD18. Analysis of cell adhesion molecules in PCPECs revealed a significant increase of ICAM-1 and VCAM-1 expression after TNFα and *S. suis *stimulation.

**Conclusion:**

Our data underline the relevance of the blood-CSF barrier as a gate for leukocyte entry into the CNS and suggest a novel transcellular migration step during the pathogenesis of bacterial meningitis.

## Background

Bacterial meningitis is still an important cause of mortality and morbidity despite advances in antimicrobial therapy [1,2]. Especially, the exact role of the blood-cerebrospinal fluid (CSF) barrier, which is constituted by epithelial cells of the choroid plexus (CP), in bacterial meningitis is under investigation [3,4]. Important functions of the CP are maintaining homeostasis in the CNS, CSF secretion and participation in neurohumoral brain modulation and neuroimmune interaction [5,6].

*Streptococcus suis *(*S. suis*) is a swine and emerging human pathogen causing a wide range of infections like meningitis and septicaemia [7]. *S. suis *has been suggested to enter the brain via the blood-CSF barrier. In fact, lesions have been observed at the CP in natural and experimentally induced cases of *S. suis *meningitis in pigs and mice [8-10]. In an inverted Transwell filter model of primary porcine CP epithelial cells (PCPECs) *S. suis *invades PCPECs specifically from the basolateral side in a capsule-dependent manner [4]. Furthermore, after apical infection of PCPECs with *S. suis*, tight junction function, morphology and protein expression is significantly altered [3,11].

Inflammatory activation of epithelial and endothelial cells, e.g. after bacterial infection, induces the release of interleukin-8 (IL-8) and other chemokines that recruit polymorphnuclear neutrophils (PMNs), which transmigrate across the cellular barriers and build the first line of defence to subcellular spaces [12,13]. For endothelial cells two possible routes for leukocyte transmigration have been described: paracellular and transcellular [14,15]. Ling et al. have reported that monocytes traverse epiplexus cells by a process called emperipolesis, whereby monocytes migrate through the epithelial cells [16]. In contrast, for PMNs only data for paracellular transmigration through epithelia exist so far [12,13].

The molecular events of a transcellular pathway involve a rather complicated mechanism wherein a zipperlike model of junctional disruption is easy to envision. Many studies may undervalue the frequency of transcellular events since they appear in very close proximity to the junctions and thus might be mistaken for paracellular migration [17,18]. Thus, the visualization of a leukocyte migrating through endothelial cytoplasm very close to, but distinct from, the junctional area requires advanced ultrastructural technical settings.

The molecular mechanisms employed by PMNs to cross endothelia and epithelia have been intensively investigated. So far only a few molecules have been found to be involved in the transmigration across epithelial monolayers; these include the leukocyte α_M_β_2_-integrin CD11b/CD18 and the leukocyte and epithelial integrin-associated glycoprotein CD47 [12,19]. In comparison, rolling PMNs adhere firmly to endothelium via leukocyte α_L_β_2_-integrin CD11a/CD18 (leukocyte function-associated antigen-1, LFA-1) and CD11b/CD18, which bind to adhesion molecules such as intercellular adhesion molecule-1 (ICAM-1) [20].

As the CP epithelium is a potential entry point for PMNs during bacterial meningitis, we here analyzed the transmigration process of PMNs through PCPEC monolayers after infection with *S. suis*. Analyzing the traversal route of PMN, we found that paracellular migration stops in front of a tight junction. Strikingly, the epithelial cell layer forms funnel-like structures protruding from the apical membrane, which are regularly found adjacent to PMNs. We propose a new mechanism of PMN transmigration in epithelial cells, whereby PMNs transmigrate through these funnels to finally overcome the epithelial barrier via a final transcellular step.

## Methods

### Bacterial strains and growth conditions

*S. suis *serotype 2 virulent strain 10 and its isogenic non-encapsulated mutant strain 10cpsΔEF, named strain 10 Δcps in this study, were kindly provided by H. Smith (DLO-Institute for Animal Science and Health, Lelystad, The Netherlands) [21]. Bacteria were maintained and inoculated as previously described [4]. Bacteria were washed twice in phosphate-buffered saline (PBS) (pH 7.3) and adjusted to an optical density at 600 nm (OD_600_) of 0.65. This stock solution had approximately 2 × 10^8 ^colony-forming units (CFU)/ml and was further diluted in fresh culture medium without antibiotics for the experiments.

### Preparation and cultivation of PCPECs on inverted Transwell filters

Epithelial cells from porcine CP were obtained by a modified preparation basically as described previously [22]. For inverted cell cultures the cells were seeded on laminin- (Sigma, Deisenhofen, Germany) coated Transwell filters (pore diameter 3.0 μm, 0.33 cm^2^; PET membrane, Falcon, BD, Le Pont De Claix, France), which were flipped over and placed in a medium-flooded 12-well plate as described recently [4].

### Measurement of transepithelial electrical resistance (TEER)

Confluence of PCPEC monolayers and barrier properties were documented by measuring TEER. TEER was measured using an epithelial tissue voltohmmeter (EVOM^®^, World Precision Instruments, Sarasota, FL, USA) and an STX-2 electrode system. PCPEC inverted cultures were used when TEER values reached more than 180 Ω × cm^2^. In PMN transmigration experiments, TEER was monitored over a range of 4 h. Resistance values of cells in medium alone were used as negative control values and stayed above 180 Ω × cm^2 ^during all experiments.

### Determination of paracellular permeability

As an independent measure of paracellular permeability of CP epithelium monolayers, the passage of Texas Red-labelled dextran (MW 3000; Sigma, Deisenhofen, Deutschland) across cell monolayers in the basolateral-to-apical direction was determined during PMN transmigration experiments. Texas Red-dextran (TR-dextran, 100 μg/ml) was loaded into the upper compartment during the incubation period. At indicated time intervals samples from the lower compartment were collected and fluorescence was measured in duplicates in a Tecan Infinite M200 Multiwell reader (Tecan, Switzerland). TEER and permeability measurements were performed with the same cultures as PMN transmigration.

### Isolation of PMNs

For the PMN transmigration assays blood was taken from freshly slaughtered pigs at the abattoir. PMNs were isolated from non-coagulated citrate blood by Percoll density sedimentation according to the manufacturer's instructions (Biochrom, Berlin, Germany). Contaminating erythrocytes were lysed with NH_4_Cl on ice. PMNs were resuspended in culture medium at a cell density of 1 × 10^7^/ml. For transmigration assays PMNs were loaded with the fluorochrome 2',7'-bis-(2-carboxyethyl)-5-(and-6)-carboxyfluorescein, acetomethyl ester (BCECF-AM; Molecular Probes, Eugene, OR, USA) according to the manufacturer's instructions.

### Stimulation of PCPECs and PMNs

For transmigration experiments inverted PCPEC cultures were used when TEER had reached 180 Ω × cm^2^. PCPECs were stimulated with either TNFα from the apical and basolateral side (10 ng/ml) for 24 h or basolaterally (blood-side) infected with *S. suis *strain 10 or strain 10 Δcps [multiplicity of infection (MOI): 10] and hereafter incubated for 2 h at 37°C and 5% CO_2_. After the incubation period penicillin/streptomycin (100 U/ml/100 μg/ml) was added to the upper and lower compartment of the Transwell filter to inhibit further extracellular bacterial growth and therefore to prevent cytotoxic effects. PMN transmigration assays were performed after an additional 22 hours on the following day. In a second set of experiments we pre-incubated PMNs with *S. suis *strain 10 or strain 10 Δcps (MOI 10) for 1 h and hereafter performed with pre-stimulated PMN transepithelial migration assays in antibiotic-containing medium (to prevent further bacterial growth) as described below.

### PMN transepithelial migration assay

For transepithelial migration assays BCECF-AM-loaded PMNs were added to the upper Transwell filter compartment (blood-side) of control, TNFα or *S. suis *stimulated cells in a PMNs:PCPECs ratio of 10:1. As chemoattractant IL-8 (10 ng/ml) was used in indicated samples and added to the lower Transwell filter compartment (CSF-side) 30 min before starting the transmigration experiments. After 4 hours of transmigration the Transwell filter inserts were removed and the 24-well plates were centrifuged (5 min, 300 × g) to ensure that all PMNs are attached to the bottom of the wells. The supernatants were collected for permeability measurements. The PMNs were washed once with HBSS with Ca^2+^/Mg^2+ ^and again centrifuged (5 min, 300 × g). Transmigrated PMNs were lysed by 1% Triton X-100 in PBS and quantified by fluorescence measurement with a Tecan 200 M Infinite Multiwell reader (Tecan, Switzerland) in relation to an internal standard. For antibody blocking experiments BCECF-AM loaded PMNs or PCPECs were pre-incubated with antibodies as indicated for 30 min at room temperature or at 37°C/5% CO_2_, respectively. Hereafter, a transmigration assay was performed as described above. Antibodies specific to porcine epitopes (Table 1) were selected referring to the analyses of the "Third International Workshop on Swine Leukocyte Differentiation Antigens" [23,24]. Cross reactivity of anti-CD47 has been described by Shahein et al. [25].

**Table 1:** Antibodies used for inhibition studies, flow cytometry and immunofluorescence.

Antibody	Clone	Species	Concentration	Company
**Primary antibodies**				

**IgG_1_**, κ Isotype	MOPC-31C	Mouse	20 μg/ml (inhibition) 1:10, 10 μl/test (FC)	BD Pharmingen (Heidelberg, Germany)

**IgG_2b_**, κ Isotype	MPC-11	Mouse	20 μg/ml (inhibition) 1:10, 10 μl/test (FC)	BD Pharmingen (Heidelberg, Germany)

**CD11a**, IgG_2b_	BL2F1	Mouse	20 μg/ml (inhibition) 1:10, 10 μl/test (FC)l	BD Pharmingen (Heidelberg, Germany)

CD11R3, IgG_1_ = **CD11b**	2F4/11	Mouse	20 μg/ml (inhibition) 1:10, 10 μl/test (FC)	Serotec (Oxford, UK)

**CD18**, IgG_1_	PNK-I	Mouse	20 μg/ml (inhibition) 1:10, 10 μl/test (FC)	BD Pharmingen (Heidelberg, Germany) or Serotec (Oxford, UK)

**CD47**, IgG_2b_	BRIC126	Mouse	20 μg/ml (inhibition) 1:10, 10 μl/test (FC)	Serotec (Oxford, UK)

**CD49d**, IgG_1_	HP2/1	Mouse	20 μg/ml (inhibition) 1:10, 10 μl/test (FC)	Serotec (Oxford, UK)

**CD49e**, IgG_1_	VC5	Mouse	20 μg/ml (inhibition) 1:10, 10 μl/test (FC)	BD Pharmingen (Heidelberg, Germany)

**Occludin**		Rabbit	1:250 (IF) 1 μg/ml	Zymed Laboratories (South San Franscisco, CA, USA)

**ZO-1**		Rabbit	1:250 (IF) 1 μg/ml	Zymed Laboratories (South San Franscisco, CA, USA)

**SWC3a-FITC**	74-22-15	Mouse	1:10 (IF) 10 μl/Test	Southern Biotech (Birmingham, AL, USA)

**Secondary antibodies**				

**Alexa fluor^® ^594 goat anti chicken**		Goat	1:1000 (IF)	Molecular Probes (Eugene, OR, USA)
**Goat anti mouse-PE**		Goat	1:10, 10 μl/test (FC)	Southern Biotech (Birmingham, AL, USA)

SWC: swine workshop cluster

### Flow Cytometry

Flow cytometric analyses was performed to test the specificity of antibodies, which were used in inhibition studies, and to investigate the expression patterns of PMN surface markers before and after transmigration. For this purpose, PMNs were stained with various antibodies according to manufacturer's instructions (Table 1). The PMN populations showed high and homogeneous integrin expression with regard to all tested integrins antibodies (data not shown). Integrin regulation before and after PMN transmigration was measured by analyzing the mean fluorescence intensities. To study the specific effect of IL-8, TNFα and *S. suis *and the transmigration itself on the integrin expression, two different additional analyses were performed. In the first, PMNs were analyzed that had transmigrated towards an IL-8 gradient (10 ng/ml in the lower compartment) through control or *S. suis *(MOI 10) or TNFα (10 ng/ml) stimulated PCPEC. In the second, PMNs were used that had not yet transmigrated, but which were stimulated in non-tissue culture-treated 24-well plates (employing 0, 6.7 or 10 ng/ml IL-8). After 4 h of transmigration or incubation with *S. suis*, TNFα and IL-8, the PMNs were collected and divided into tubes. After centrifugation (5 min, 300 × g) the PMNs were incubated with the primary antibodies for 12 min. Then the cells were washed twice with cell culture medium and incubated with the secondary antibody (goat anti mouse-PE) for 7 min followed by two washing steps. Flow cytometry was performed using a FACScan (Becton Dickinson, USA) with appropriately set light scatter gates.

### Immunofluorescence

Confluent PCPECs were grown on inverted Transwell filters, stimulated with *S. suis *or TNFα and co-cultured with PMNs as described above. After 4 h of transmigration towards a gradient of IL-8, the cells were washed, fixed and permeabilized as described previously [3]. Subsequently, the cells were washed with PBS and incubated overnight at 4°C with the primary antibodies (Table 1) to stain the TJ proteins. On the following day the cells were washed again, incubated for 60 min with the secondary antibody (Alexa fluor^® ^594 goat anti-chicken), with Phalloidin Alexa fluor^® ^660 for staining the actin cytoskeleton and with 4'-6-diamidino-2-phenylindole dihydrochloride (DAPI) (1:25.000) for staining nuclei. PMNs were labelled with the granulocyte-monocyte marker SWC3a-FITC (1:10 in PBS) (Southern Biotech, Birmingham, AL, USA) for 30 min. After washing the cells three times with PBS the filters were embedded in ProLongAntifadeReagent (Invitrogen, Karlsruhe, Germany). Images were acquired with Zeiss Apotome^® ^and Axiovision software (Carl Zeiss, Jena, Germany) using a 63×/1.4 objective lens. The image acquisition was carried out using the Zeiss scanning software Axiovison 4.6 and Axiovison module Inside 4D. Assays were performed in triplicates for each value and repeated at least four times.

### Transmission electron microscopy (TEM)

PCPECs were grown on inverted Transwell filters, stimulated and co-cultured with PMNs as described above. After a 4-h coincubation period, the cells were washed once with culture medium, twice with Dulbecco's-PBS and hereafter fixed with 2% glutaraldehyde (Polyscience, Warrington, PA, USA) in D-PBS, pH 7.4, for 24 h at 4°C. The Transwell filter membranes were cut out of the insert and washed three times with D-PBS and post-fixed with 1% osmiumtetroxide. After another three washes with D-PBS the samples were dehydrated by a graded ethanol series (30%, 50%, 70%, 90%, 96% for 15 min each, 2 × 99% for 30 min each) and two washes with propylenoxide. During the 70% ethanol step of the graded ethanol series, the specimens were incubated in saturated uranyl acetate. After completion of dehydration, the preparations were embedded in Araldite 502 (Sigma-Aldrich) at 60°C for 48 h. Ultrathin sections were prepared on a Leica FCR Ultracut ultramicrotome and stained with lead citrate. Sections were examined using a Zeiss EM 10 electron microscope.

### Scanning electron microscopy

Samples were fixed with 2.5% glutaraldehyde in cacodylate buffer, postfixed with 1% osmium tetroxide in phosphate-buffered saline, dehydrated in a graded series of ethanol and critical-point-dried using CO_2_. Finally, the samples were sputter-coated with a layer of 7 nm gold/palladium (Bal-Tec MED 010) and examined at 20 kV accelerating voltage in a Hitachi S-800 field emission scanning electron microscope.

### Quantitative real-time PCR

After treatment with *S. suis *[strain 10 and strain 10cpsΔEF (short: strain 10 Δcps)] or TNFα (10 ng/ml) for 2 and 4 h inverted PCPEC monolayers were washed with PBS and total cellular RNA was extracted using the RNeasy mini kit (Qiagen, Hilden, Germany) modified for inverted cell cultures. For every stimulus, three Transwell filters were used. Briefly, Transwell filter membranes were cut out of the insert and transferred into 48 wells. The cells on the filter membrane were lysed by resuspending them in 100 μl of RLT (ready to load) buffer. Lysed cells of every triplicate were collected and filled to 350 μl with RLT buffer and homogenized by QIAshredder (Qiagen, Hilden, Germany) followed by RNA extraction. Contaminating DNA was digested with the RNase-free DNase I (Roche, Mannheim, Germany) for 60 min at room temperature. DNase was inactivated by 1 min at 72°C. After spectrophotometrical determination of the RNA concentration using a NanoDrop (Thermo Scientific, Wilmington, USA) 145 - 300 ng of total RNA was reverse-transcribed with the SuperScript™ III First-Strand Synthesis System (Invitrogen, Karlruhe, Germany) for real-time PCR according to the manufacturer's instructions. Quantitative real-time PCR was performed with 2× Quanti Tect SYBR Green PCR Master Mix (Qiagen, Hilden, Germany) and the 7900HT Fast Real-Time PCR System (Applied Biosystems, Darmstadt, Germany). cDNA quantities were measured as critical threshold (C_T_) values, which were then normalized using simultaneously measured β-actin levels (ΔC_T_). Final ΔΔC_T _values were obtained by comparing bacteria-stimulated or TNFα-treated cells with unstimulated cells using PCPECs from three different preparations. Primers used were previously described [26] (Table 2). The specificity of PCR products was checked by agarose gel electrophoresis (data not shown).

**Table 2:** Primers used for quantitative real-time PCR

Gene	Primer	Sequence (5' → 3')
ICAM-1	forward	CACAGGCCGCCACTAACAA
ICAM-1	reverse	GGTTCCATTGATCCAGGTCTT
VCAM-1	forward	GCACGAGCTTCCTGAGCACTT
VCAM-1	reverse	CTGTGTGACGAGGAAACAATG
β-actin	forward	TCCAGAGGCGCTCTTCCA
β-actin	reverse	CGCACTTCATGATCGAGTTGA

ICAM, intracellular adhesion molecule; VCAM, vascular cell adhesion molecule

### Measurement of cell viability

Cell vitality was measured using the Live/Dead^® ^Viability/Cytotoxicity Kit for mammalian cells (Molecular Probes, Göttingen, Germany) according to the manufacturer's instructions. The results were photodocumented by fluorescence microscopy. In all experiments no cytotoxicity was observed (data not shown).

### Statistical analysis

All data are expressed as means ± standard deviation (SD) as indicated. TEER, dextran-flux, FACS analysis and real-time PCR data were analyzed by Student's *t *test respectively, PMN transmigration data by an analysis of variance (ANOVA) with repeated measurements and the method of *compound symmetry *was performed. *P*-values for post-hoc test were adjusted by the method of Tukey-Kramer. The procedure of MIXED of SAS was used. A *P *value of < 0.05 was considered significant. All assays were repeated at least three times. Texas Red-dextran flux data were expressed as percent of tracer in the basolateral compartment.

## Results

### IL-8 and PMN transmigration itself do not alter PCPEC barrier function

**Figure 1:**
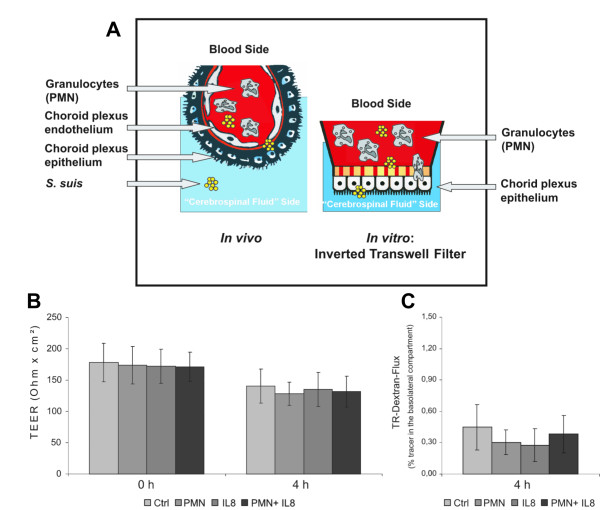
**Effects of IL-8 and PMN transmigration on PCPEC barrier function**. The inverted Transwell system used to investigate PMN transmigration is displayed in (A). (B, C) Effects of IL-8 or PMN transmigration on barrier function were measured by TEER (B) and TR-dextran flux (C) of PCPEC monolayers. IL-8 (10 ng/ml) was applied to the lower compartment, and PMNs (PCPECs:PMNs ratio 1:10) to the apical compartment of inverted PCPEC cultures or PMNs and IL-8 in combination. The TEER results are expressed as absolute TEER values (Ω × cm^2^) and were measured before and after 4 h of PMN transmigration. TR-dextran flux was determined in the basolateral-to-apical direction (upper to lower compartment) after 4 h of incubation and is expressed as percentage of tracer in the basolateral compartment. Data are shown are mean ± SD for four independent experiments, each performed in triplicate.

For human brain microvessel endothelial cells (HBMECs), it has been described that PMN transmigration leads to a loss of blood-brain barrier function, which is determined by a drop in TEER and enhanced paracellular permeability [27]. To determine whether this also applies for the blood-CSF barrier we measured TEER and paracellular TR-dextran flux in bacterially stimulated and unstimulated PCPEC in our previously described inverted Transwell filter system [4] (Figure 1A). Contrary to the results observed with HBMEC no significant changes in barrier function could be observed following PMN transmigration through unstimulated PCPECs in presence or absence of IL-8 for up to 4 hours. Figure 1B shows no significant decrease of TEER during the incubation time of 4 hours in all tested conditions. In parallel, no significant increase in paracellular permeability was induced by IL-8, PMN or IL-8/PMN coincubation in comparison to control cells (Figure 1C).

### *S. suis *induces alteration barrier function in PCPECs in the presence of PMNs

**Figure 2:**
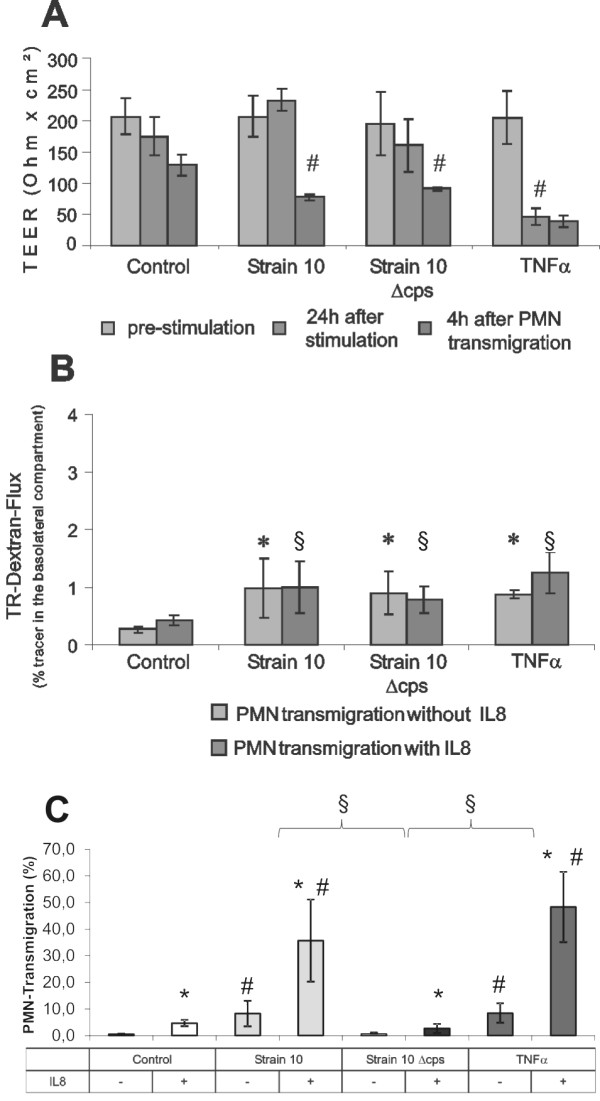
**Effect of *S. suis *and TNFα on PMN transmigration through PCPEC and barrier function**. (A, B, C) PCPECs were infected with *S. suis *strain 10 and the non-encapsulated mutant strain 10 Δcps (MOI 10) or stimulated with TNFα (10 ng/ml) as described in Methods. PMNs were applied after 24 h of stimulation to the basolateral side of PCPEC. Effects on barrier function were measured by TEER (A) and TR-dextran flux (B) of PCPEC monolayers. The TEER results are expressed as absolute TEER values (Ω × cm^2^). (A) For simplification only TEER values of IL-8-treated PCPECs are shown because they did not differ from TEER values of PCPEC cultures without IL-8. TEER was measured before stimulation, 24 h later that was directly before PMN transmigration and after 4 h of PMN transmigration. #, P value of < 0.001, compared to time point before.(B) The TR-dextran flux was measured in the basolateral-to-apical direction (upper to lower compartment) with or without S. suis infection, respectively, and TNFα stimulation and is expressed as percentage of tracer in the basolateral (upper) compartment. TR-dextran flux was determined after 4 h of PMN transmigration. In indicated samples IL-8 (10 ng/ml) was used as chemoattractant. Data are shown are mean ± SD for four independent experiments, each performed in triplicate. *, P value of < 0.05 compared to corresponding control PCPECs without IL-8 stimulation. §, P value of < 0.05 compared to corresponding control PCPECs with IL-8 stimulation. (C) BCECF-loaded PMNs were applied after 24 h of stimulation to the basolateral side of inverted PCPEC in a PCPECs:PMNs ratio of 1:10. In indicated samples IL-8 (10 ng/ml) was used as chemoattractant. The number of PMNs was fluorometrically measured after 4 h of transmigration. PMN transmigration is expressed% PMN transmigration from totally applied PMNs. Data are shown as mean ± SD for four independent experiments, each performed in triplicate. *, P value of < 0.0001 compared to PCPEC without IL-8; #, P value of < 0.0001, compared to corresponding control PCPEC +/- IL-8; §, P value of < 0.0001 compared between different stimuli.

We were next interested in analyzing the influence of PMNs on PCPECs that had been infected with bacteria. Since previous studies have demonstrated an increased permeability caused by TNFα in cellular barriers, especially for brain endothelial cells [28] and PCPECs in a standard Transwell system [26], we used this cytokine for comparison with bacteria-infected PCPECs in the inverted Transwell filter cultures. In transmigration assays PCPECs were incubated with or without IL-8 in the lower filter compartment as a chemoattractant for PMN migration. TEER data are shown for simplicity only for IL-8-treated cells (Figure 2A), but results of experiments without IL-8 were not different indicating that IL-8 has no significant impact on barrier function (data not shown). Figure 2A shows a significant decrease of TEER to 24% of initial values after 24 h of TNFα stimulation. This goes in parallel with a significant 3-fold increase in paracellular permeability (Figure 2B). No significant changes in TEER were observed within the first 24 hours under control conditions or after infection with *S. suis *strain 10 or the non-encapsulated mutant 10 Δcps. In contrast, after a 4-hour course of PMN transmigration we observed a significant drop of TEER after bacterial infection. However, the significant TEER drop caused by TNFα stimulation alone did not change significantly after PMN migration. Paracellular TR-dextran flux remained low, but was nevertheless significantly enhanced by *S. suis *strain 10 and strain 10 Δcps stimulation compared to controls, whereas bacteria- and TNFα-induced paracellular fluxes did not differ significantly from each other. In line with the TEER analyses, no differences in TR-dextran fluxes could be observed for IL-8-treated and control cells.

### *S. suis *and TNFα induce PMN transmigration through PCPECs

After bacteria have entered the brain, the influx of leukocytes from the blood into the CNS is a crucial step in the development of meningitis. In our recent study we investigated transmigration of the well-encapsulated *S. suis *wild-type strain 10 and its capsule-deficient isogenic mutant strain 10 Δcps, and identified the capsule as a critical factor for adhesion to and invasion into PCPECs [4]. Infection of PCPECs from the physiologically relevant basolateral side with *S. suis *strain 10 (MOI 10) or apical and basolateral stimulation with TNFα (10 ng/ml) leads to significant PMN transmigration (Figure 2C). In contrast, infection with the capsule-deficient mutant strain 10 Δcps did not cause an increased PMN migration rate; in fact this was significant lower than in *S. suis *strain 10 or TNFα-treated cells, and comparable to that of control cells. In control cells as well as in *S. suis *or TNFα-treated cells the transmigration rate could be further significantly increased by the chemoattractant IL-8. In a second set of experiments we infected PMNs directly with *S. suis *strain 10 or strain 10 Δcps (MOI 10) and subsequently performed PMN migration assays with IL-8-treated cells. We again observed a significantly lower transmigration rate after stimulation with the capsule-deficient strain, and for both *S. suis *strains a significant reduction of PMN transmigration rate compared to uninfected control PCPECs (data not shown). Moreover, TEER and paracellular TR-dextran flux of *S. suis*-infected cells were not significantly compromised compared to uninfected control PCPECs (data not shown).

### Tight junction and actin cytoskeleton morphology after PMN transmigration

**Figure 3:**
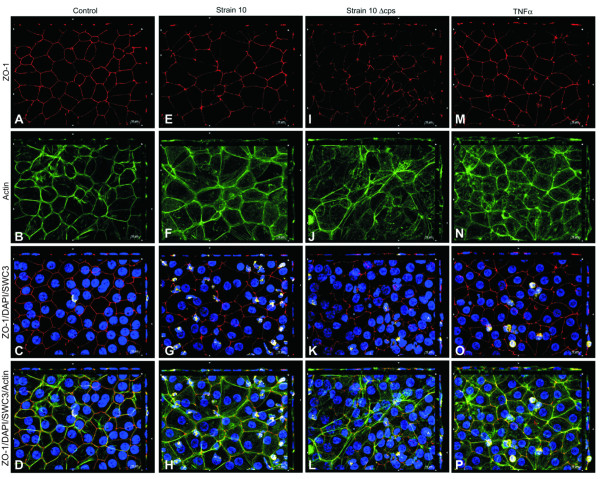
***S. suis *and TNFα alter the morphology of the tight junction protein ZO-1 and induce actin stress fiber formation after the transmigration of PMNs across PCPECs**. (A-P) *En face *Apotome^® ^microscopy of (A-D) control, (E-H) *S. suis *strain 10, (I-L) strain 10 Δcps and (M-P) TNFα-stimulated PCPEC monolayers stained for ZO-1 (red), actin (phalloidin, green) and nuclei (DAPI, blue) after PMN (yellow) transmigration. Top and side of each panel is a cross section through the z-plane of multiple optical slices. *S. suis *and TNFα-stimulated PCPECs showed an altered tight junction morphology and increased actin stress fiber formation. This figure is a representative example of three independent experiments that all gave similar results. Scale bar, 10 μm.

**Figure 4:**
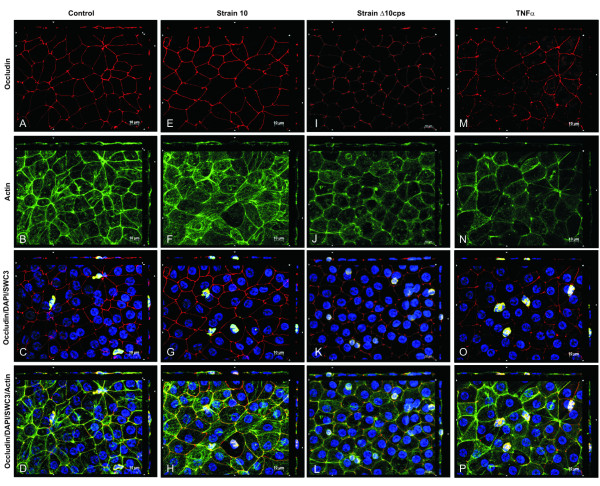
***S. suis *and TNFα alter the morphology of the tight junction protein occludin and induce actin stress fiber formation after the transmigration of PMNs across PCPECs**. (A-P) En face Apotome microscopy of control (A-D), *S. suis *(strain 10: E-H, strain 10 Δcps: I-L) and TNFα- (M-P) stimulated PCPEC monolayers stained for occludin (red), actin (phalloidin, green) and nuclei (DAPI, blue) after PMN (yellow) transmigration. Top and side of each panel is a cross section through the z-plane of multiple optical slices. This figure is a representative example of three independent experiments that all gave similar results. Scale bar, 10 μm.

The next set of experiments was performed to test if changes in barrier properties induced by *S. suis*, TNFα and PMN correlate with morphological changes in TJ and actin cytoskeleton distribution. *S. suis*-infected and especially TNFα-stimulated PCPECs co-incubated with (Figure 3, 4) or without PMNs (data not shown) showed a fuzzy and blurred distribution pattern of ZO-1 (Figure 3E, I, M) and occludin changes (Figure 4E, I, M), whereas control cells incubated with or without PMNs displayed a more clear and smooth protein expression at the cell borders (Figure 3A, 4A (and data not shown)). Stimulation of PCPECs especially with TNFα led to a decreased staining pattern of the TJ proteins ZO-1 and occludin, indicating stronger alteration and partial loss of the TJ proteins, which goes in line with our previously published results in the standard Transwell system [3,26]. The phalloidin-stained actin cytoskeleton of unstimulated control cells incubated with or without PMNs showed very clear structures and well-defined perijunctional apical actin rings (Figure 3B, 4B (and data not shown)). After co-stimulation of PCPECs with bacteria or TNFα in the presence or absence PMNs the appearance of the actin cytoskeleton changed from clear structures to a diffuse actin staining in the apical and basolateral cell compartment, with reduced colocalization with ZO-1 or occludin, and stress fiber formation (Figure 3F, J, N; Figure 4F, J, N (and data not shown)). Stress fibers appeared partly in cabled bundles in the basolateral cell compartment.

### Transcellular PMN traversal through PCPECs

**Figure 5:**
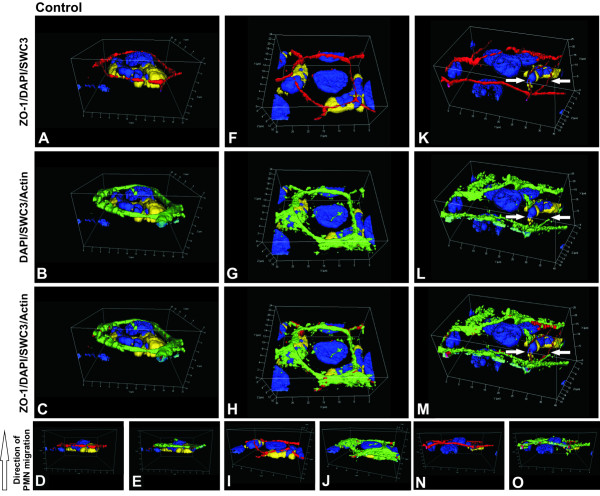
**PMN traversal across inverted control PCPEC cultures**. PCPEC monolayers were stained for ZO-1 (red), actin (phalloidin, green), nuclei (DAPI, blue) and PMNs (SWC3a-FITC, yellow). Three-dimensional immunofluorescence images of control PCPECs co-cultured with PMNs were reconstructed from 0.3 μm Apotome^® ^optical sections, using Zeiss software Inside 4D. Views from above (on the apical cell side) show different phases of PMN migration. The lower row represents side views (D, E, I, J, N, O). A-E. Transcellular PMN migration in distance to ZO-1. F-J. PMN below tight junctions and actin ring. K-O. PMN migration stops below altered tight junctions with altered ZO-1 strands (white arrows). This figure is a representative example of three independent experiments that all gave similar results. Scale bar, as indicated.

**Figure 6:**
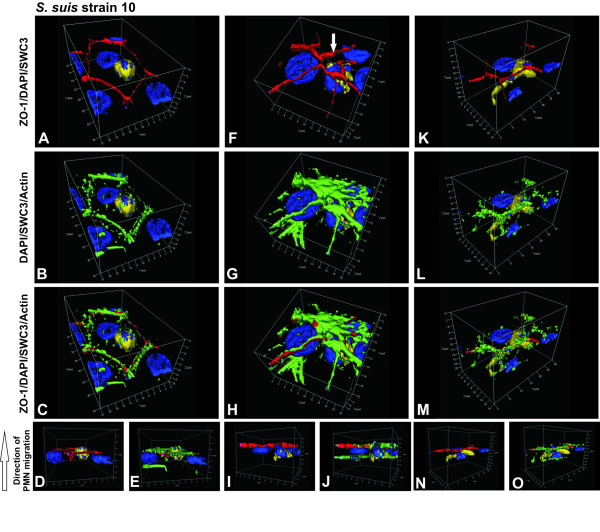
**PMN traversal across *S. suis *strain 10-infected PCPECs**. PCPEC monolayers were stained for ZO-1 (red), actin (phalloidin, green), nuclei (DAPI, blue) and PMNs (SWC3a-FITC, yellow). Three-dimensional immunofluorescence images of *S. suis *strain 10-infected PCPECs co-cultured with PMNs were reconstructed from 0.3 μm Apotome optical sections, using Zeiss software Inside 4D. Views from above (on the apical cell side) show different phases of PMN migration. The lower row represents side views (D, E, I, J, N, O). (A-E) PMN transmigration through the center of a PCPEC cell body. (F-J) PMN migration with beginning of ZO-1 alteration at a tricellular corner (white arrow). (K-O) PMN migration along the intercellular junction ZO-1. This figure is a representative example of three independent experiments that all gave similar results. Scale bar, as indicated.

**Figure 7:**
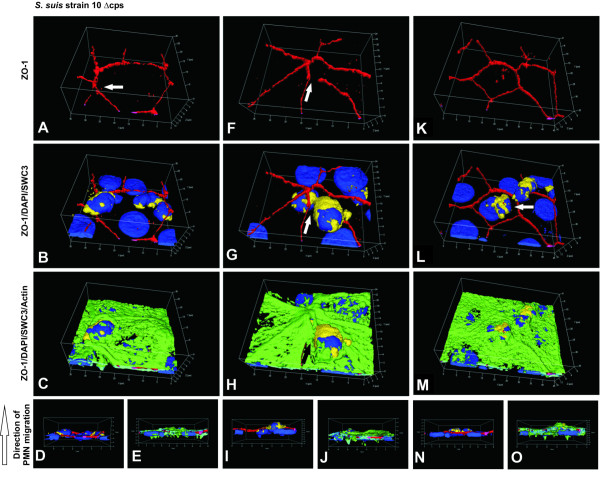
**PMN traversal across *S. suis *strain 10 Δcps-infected PCPECs**. PCPEC monolayers were stained for ZO-1 (red), actin (phalloidin, green), nuclei (DAPI, blue) and PMNs (SWC3a-FITC, yellow). Three-dimensional immunofluorescence images of *S. suis *strain 10 Δcps-infected PCPEC co-cultured with PMNs were reconstructed from 0.3 μm Apotome optical sections, using Zeiss software Inside 4D. Views from above (on the apical cell side) show different phases of PMN migration. The lower row represents side views (D, E, I, J, N, O). (A-E) PMN below TJ (right) and above TJ (left) and in the middle of a PCPEC (trancellular). The region where the left PMN migrated shows loose and diffuse actin staining at the upper PCPEC pole but the ZO-1 strands appears intact (white arrow). (F-J) PMN migration close to an interrupted ZO-1 strand (white arrow). (K-O) Transcellular PMN migration (left PMN, white arrow). The actin cytoskeleton is disarranged where the PMN exits the PCPEC (O). This figure is a representative example of three independent experiments that all gave similar results. Scale bar, as indicated.

**Figure 8:**
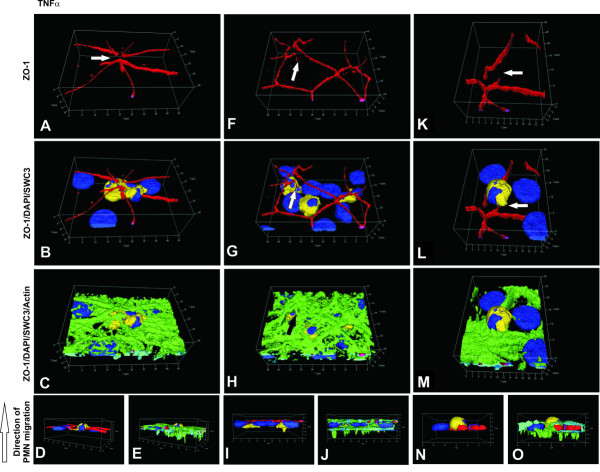
**PMN traversal across TNFα-stimulated PCPECs**. PCPEC monolayers were stained for ZO-1 (red), actin (phalloidin, green), nuclei (DAPI, blue) and PMNs (SWC3a-FITC, yellow). Three-dimensional immunofluorescence images of TNFα-stimulated PCPECs co-cultured with PMNs were reconstructed from 0.3 μm Apotome optical sections, using Zeiss software Inside 4D. Views from above (on the apical cell side) show different phases of PMN migration. The lower row represents side views (D, E, I, J, N, O). (A-E) PMN located below a multicellular corner with a gap in the ZO-1 structure (white arrow). (F-J) Transcellular (central PMN) PMN and migration close to TJ (left PMN). The right PMN is located directly beyond an intact TJ strand, the left one is close to disrupted ZO-1 (white arrow). (K-O) PMN migration close to an interrupted TJ strand (white arrow). This figure is a representative example of three independent experiments that all gave similar results. Scale bar, as indicated.

Next we performed detailed and sequential microscopic studies to elucidate the PMN transmigration process. Three dimensional immunoflourescence analyses show PMNs in different phases of PMN migration through unstimulated cells (Figure 5), *S. suis *strain 10 (Figure 6), *S. suis *strain 10 Δcps (Figure 7) and TNFα- (Figure 8) stimulated cells. In Figure 5A-E and 6A-E the PMN is located in the centre of the cell that it has almost completely crossed. The PMN is also seen in a position relative to the PCPEC cell nucleus. An elongated PMN below a TJ strand is shown in uninfected cells (Figure 5F-J). Figure 6F-J show a PMN that still lies under the TJ strands at a tricellular corner within the PCPEC monolayer (Figure 6I, J), where ZO-1 is structurally altered (Figure 6F) and the actin cytoskeleton shows a strong condensation (Figure 6G, H). The main part of the PMN is found within the PCPEC cell level (Figure 6I-J). Figure 6K-O, indicates PMN migration along altered tight junctions, but not through them. Immunofluorescence analyses of different phases of PMN migration through *S. suis *strain 10 Δcps and TNFα-stimulated cells showed similar findings and an altered tight junction morphology close to a PMN (Figures 7, 8).

**Figure 9:**
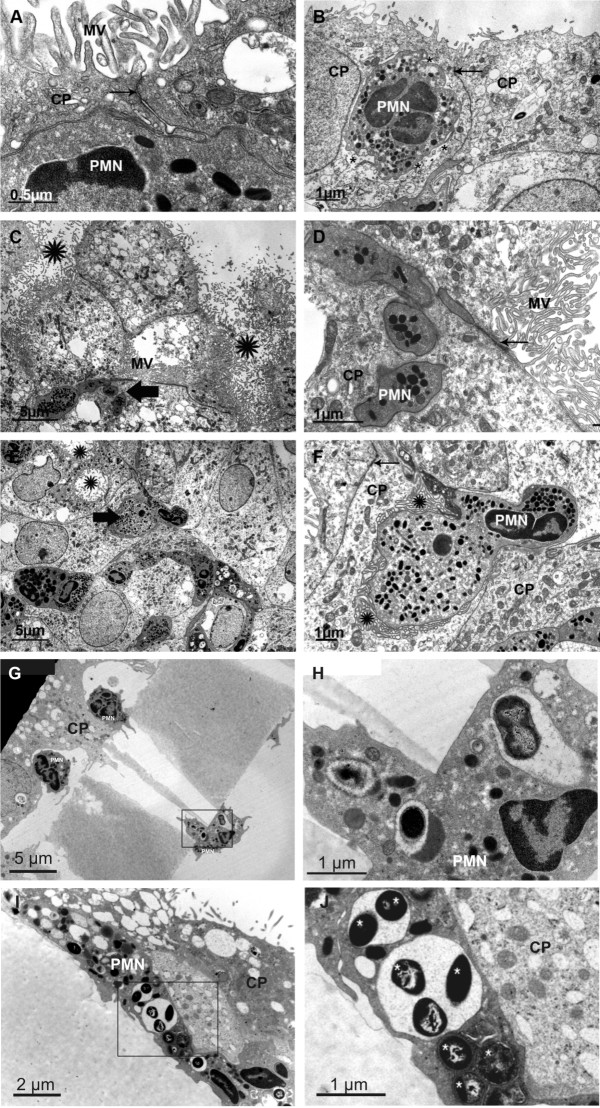
**Electron microscopic view of a PMN travelling through the CP monolayer**. (A, G, H) PCPECs were stimulated with *S. suis *strain 10, (B, I, J) *S. suis *strain 10 Δcps or (C-F) TNFα. (A) A PMN is closely associated to two epithelial cells (CP), near the tight junction (thin arrow). MV = microvilli. (B) In this case, the PMN has nearly touched the apical membrane for release of the cell into the upper compartment. The tight junction is labelled by an arrow. The space between the PMN and the epithelial cell (CP) is labelled by small asterisks. (C) Inability of PMN to open the tight junction, but in parallel, the PMN tries to enter the epithelial cell in order to reach the surface of the epithelial cell which is strongly enlarged by numerous microvilli (MV). In addition, the lower magnification in C suggests an indentation of the apical surface of the epithelial cell layer forming funnel-like structures (asterisks in C). (D) The area shown in D at higher magnification is labelled in C by a thick arrow. (E) A similar funnel is cross-sectioned in a flat section parallel to the filter (asterisks). (F) Higher magnified area labelled by a thick arrow in (E). The section runs parallel or slightly oblique to the apical surface of the monolayer. The PMN is completely surrounded by vesicular membranes which, in the REM investigation (Figure 5E), can be identified as formed by the PMN itself. Again, the tight junction (thin arrow) remains closed and unaffected. (G) PMN harbouring encapsulated *S. suis *strain 10 in vacuoles. (H) Enlargement of the marked section of G; bacteria are marked with a asterisks. (I) PMN harbouring non-encapsulated *S. suis *strain 10 Δcps in vacuoles (white marks). (J) Enlargement of the marked section of I; bacteria are marked with an asterisk. Scale bar, as indicated.

**Figure 10:**
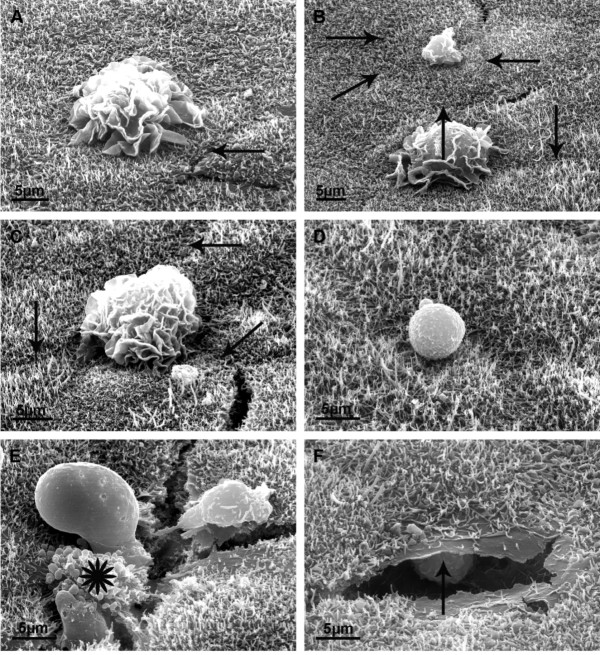
**Scanning electron microscopical analysis and serial sections of PMNs transmigrating through PCPEC monolayers**. Arrows mark cell borders. (A-C) The cell in A and the lower cell in B could have been overcome the barrier in the middle of an epithelial cell, apart from the cell border, whereas the smaller cell in B, and also the cell in C, could have just transmigrated between two epithelial cells. (D) A PMN lies in an indentation in the middle of an epithelial cell, corresponding to the funnel-like structure as described in Figures 4 and 5. (E) The vesicular structures (star) of a transmigrating PMN are shown which correspond to the surface foldings of the PMN in ultrathin sections (asterisk. Scale bar, as indicated.in Figure 4F). (F) The image shows a preparation artifact allowing a look into the space below the surface of the epithelial monolayer, where a PMN is waiting to transmigrate (arrow points to the expected direction of the cell). Here it is evident that the PMN will transmigrate transcellularly, because no cellular border can be observed.

Electron microscopical investigations were performed in order to further describe the mode of transmigration of PMNs through the PCPEC monolayer. We compared orthogradely sectioned filters with filters sectioned nearly parallel through the apical surface to allow better interpretation of cellular details during transmigration. In Figure 9A, we see a PMN directly associated with two CP cells interconnected by a tight junction (arrow). The PMN is closely squeezed between the two epithelial cells, but it appears that the cell is not able to overcome the tight junction-based barrier beneath the microvilli. In 9B, an intracellular location of the largest portion of a PMN within an epithelial cell is shown. Intracellular location in this context means that a PMN is in a vacuolar structure and not in the cytoplasm. The arrow points to the intact tight junction, which is clearly distant from the transmigrating cell. In 9C and 9D, another example is shown of the inability of a PMN to open the tight junction (arrow in 9D) but, in parallel, the PMN tries to enter the epithelial cell in order to reach the surface of the epithelial cell which is strongly enlarged by numerous microvilli. In addition, the lower magnification in 9C suggests an indentation of the apical surface of the epithelial cell layer forming funnel-like structures (asterisks in 9C). These indentations were as well lined by many microvilli proving again these membranes as apical ones. We termed these indentations funnel-like structures and assumed that they are the preferred locations where the transcellular transmigration could occur. The area shown in 9D at a higher magnification is labelled in 9C by a thick arrow. A similar funnel cross-sectioned in a flat section parallel to the filter is shown in 9E by asterisks. The area that is shown in higher magnification in 9F is labelled by a thick arrow in 9E. The section runs parallel or slightly oblique to the apical surface of the monolayer. The PMN is completely surrounded by vesicular membranes which, in the REM investigation (Figure 10E), can be identified as formed by the PMN itself. Again, the tight junction (thin arrow) remains closed and unaffected. Another striking finding was the presence of bacteria in large vacuoles within PMN during transmigration through PCPEC (Figure 9G-J). We made this observation, which suggests a traversal mechanism known as "Trojan horse", with *S. suis *strain 10 Δcps (Figure 9I,J) as well as with the wild-type strain (Figure 9G,H).

**Figure 11:**
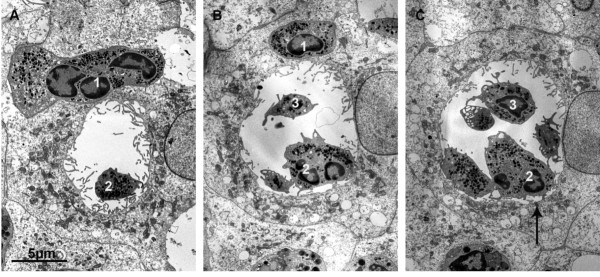
**Transcellular PMN transmigration through funnel-like indentations at the apical PCPEC membrane**. (A-C) The TEM images are serial sections in parallel to the Transwell filter of TNFα-stimulated PCPEC monolayers with an interval of 2-3 μm between them. In the middle, we have the cross-sectioned funnel lined by a seam of microvilli and rare cilia of one cell. (A) basal body of a cilium is labelled by an arrow in (B), proving that this surface is the apical surface of the epithelial cell. In this figure, identical cell profiles in the different pictures are numbered correspondingly. Scale bar, as indicated.

In scanning electron microscopic images PMNs were regularly found directly on the apical surface of the monolayer and at a distance from the intercellular clefts (Figure 10). Due to the possibility that, after completion of transmigration, the PMN can migrate over the surface of the epithelial cells, the site of former transmigration in principle cannot be determined unequivocally. However, the cell in 10A and the lower cell in 10B could have overcome the barrier in the middle of an epithelial cell, apart from the cell border, whereas the smaller cell in 10B, and also the cell in 10C, could have just transmigrated between two epithelial cells. In 10D, a PMN lies in an indentation in the middle of an epithelial cell, corresponding to the funnel-like structure described in Figure 9 and below. In 10E, the vesicular structures (asterisks) of a transmigrating PMN are shown which correspond to the surface folding of the PMN in ultrathin sections (asterisks in Figure 9F). Finally, 10F shows a preparation artefact allowing a look into the space below the surface of the epithelial monolayer, where a PMN is waiting to transmigrate (the arrow points to the expected direction of the cell). Here it seems evident that the PMN will transmigrate transcellularly, because no cellular border can be observed. In Figure 11A-C we investigated the funnel-like indentations of the cell layer of a TNFα-stimulated PCPEC and performed serial sections parallel to the Transwell filter at intervals of 2-3 μm. In the middle, we have the cross-sectioned funnel lined by a seam of microvilli and rare cilia of one cell (a basal body of a cilium is labelled by an arrow in 9I, proving that this surface is the apical surface of the epithelial cell). Obviously, PMNs take the route via the funnel to overcome the epithelial barrier. In this, identical cell profiles in the different pictures are numbered correspondingly. It is a striking observation that such funnels are regularly found adjacent to PMNs. We observed these funnel-like structures in *S. suis *or TNFα-stimulated as well as unstimulated PCPECs, but only in the presence of PMNs (data not shown).

### PMN transmigration through PCPECs is CD11b/CD18 dependent

**Figure 12:**
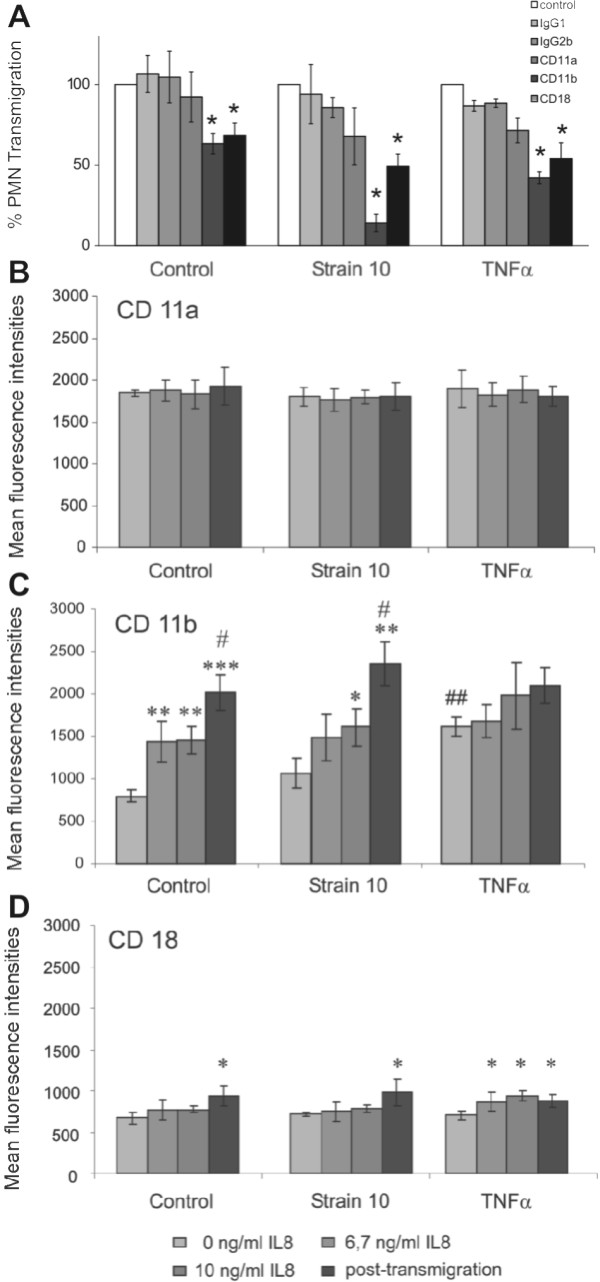
**Inhibitory effects of integrin-specific antibodies on PMN transmigration and flow-cytometric analysis of integrin expression on PMNs dependent on IL-8 and PMN transmigration after *S. suis *infection or TNFα stimulation**. (A) In blocking experiments PMNs were pre-incubated with antibodies specific to the PMN integrins CD11a, CD11b or CD18. PMN transmigration towards an IL-8 gradient (10 ng/ml) through non-stimulated and pre-stimulated PCPEC (*S. suis *strain 10 (MOI 10) or TNFα (10 ng/ml)) was analyzed hereafter as described in the Method section. Data are presented as percent of migrated PMNs not pre-incubated with antibodies. Data are shown as mean ± SD for four independent experiments each performed in triplicate. *, P value of < 0.05 compared to unstimulated, respectively, stimulated PCPEC not pre-incubated with specific antibody. (B-D) Graph show the influence of different IL-8 concentrations combined with different stimuli on CD11a, CD11b or CD18 expression pre and post-transmigration. Results are presented as mean fluorescence intensities ± SD for 4 independent experiments.***, P value of < 0.0001; **, P value of < 0.01; *, P value of < 0.05 compared to PCPECs not stimulated with IL-8, #, P value of < 0.01 compared to cells treated with 10 ng/ml IL-8, ##, P value of < 0.01 compared *S. suis*-treated and control cells.

The cellular and molecular mechanisms of PMN transmigration through epithelial cell layers are still largely under investigation. To identify the integrins that play a role in PMN transmigration from the blood side through CP epithelium, we performed inhibition studies with antibodies cross-reacting to porcine cell surface molecules, which have largely been described [23,24]. Binding of these antibodies to their respective epitopes was confirmed by FACS analyses as described in Methods. A significant effect could be detected after pre-incubation of PMNs with anti-CD11b and anti-CD18, but not with anti-CD11a (Figure 12A). Moreover we tested the inhibitory properties of antibodies specific to the α_4_-integrin CD49d (VLA-4), the α_5_-integrin CD49e (VLA-5) and the integrin-associated glycoprotein CD47 to reduce PMN transmigration. Neither anti-CD49d nor -CD49e pre-incubated with PMNs, nor anti-CD47 on epithelial cells could diminish their transmigration rate (data not shown). Combinations of anti-CD47 and anti-CD11a, -CD11b and -CD18 showed also no additive inhibitory effects (data not shown). The transmigration of PMNs primed with isotype controls was comparable to that of untreated PMNs for all conditions. To clarify if antibodies themselves compromised barrier function we also measured TEER and paracellular TR-dextran flux and found no significant influence (data not shown).

To validate the observed inhibitory effects of antibodies against CD11a, CD11b and CD18 in PMN transmigration assay we analyzed the expression patterns of these integrins on PMNs under different stimuli via flow cytometry. The presence of IL-8, which was used as chemoattractant in the assay, potentially stimulated the expression of PMN integrins [29]. Therefore, we investigated the effect of IL-8 and transmigration in principle on integrin expression in unstimulated control cells, TNFα- and *S. suis *strain 10-stimulated cells. For transmigration assays 10 ng/ml IL-8 were added to the lower compartment, which would lead to a concentration of 6.7 ng/ml in the whole well assuming an even distribution of IL-8 in the upper and lower well by diffusion. Since it was impossible to determine the exact extent of the IL-8 gradient in our experimental set-up, two different IL-8 concentrations (6.7 and 10 ng/ml) were applied, corresponding to the lowest (6.7 ng/ml) and the highest (10 ng/ml) possible concentrations. FACS analyses of PMNs after *S. suis *strain 10 Δcps stimulation was not possible due to the high background fluorescence of engulfed bacteria. During our flow cytometric analyses no significant differences between the two IL-8 concentrations were observed. The flow cytometric analyses of CD11a (Figure 12B) exhibited no differences in expression pattern before or after PMN transmigration for all tested stimuli. A significant increase of CD18 expression could be observed post-transmigration in control and *S. suis *treated cells, after IL-8 stimulation and post transmigration in TNFα-treated cells (Figure 12D). Corresponding to inhibition studies with anti-CD11b, we observed a significant upregulation of CD11b expression (Figure 12C). For all tested stimuli the expression pattern of CD47, CD49d and CD49e under IL-8 treatment or after transmigration were not altered in comparison to control cells (data not shown). This is consistent with inhibition studies, that showed no blocking effect of anti-CD47, -CD49d, and -CD49e antibodies on PMN transmigration.

### Effects of *S. suis *and TNFα stimulation on ICAM-1 and VCAM-1 mRNA expression

**Figure 13:**
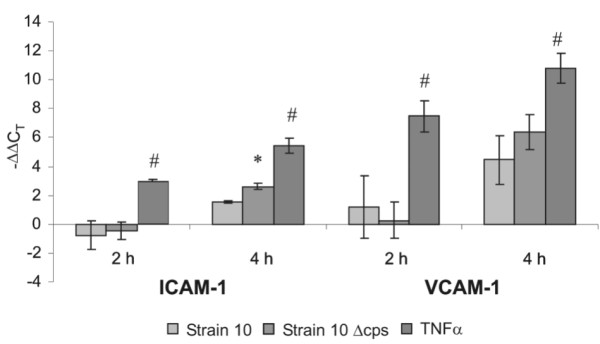
**Effect of *S. suis *and TNFα on mRNA expression of ICAM-1 and VCAM-1**. Quantitative real time-PCR analysing the effect of *S. suis *strain 10 (MOI 10), *S. suis *strain 10 Δcps (MOI 10) and TNFα (10 ng/ml) on intercellular adhesion molecule-1 (ICAM-1) and vascular cell adhesion molecule-1 (VCAM-1) mRNA expression by porcine choroid plexus epithelial cells (PCPECs). Cells had been cultured on Transwell filters in the inverted system. Bacteria were added to the basolateral side (upper compartment) of the inverted cell cultures. TNFα stimulation was performed from basolateral and apical side. -ΔΔC_T _values are based on β-actin standardization as well as on comparison to unstimulated control cells. Positive -ΔΔC_T _values indicate an mRNA upregulation. Results are presented as means ± SD for at least 3 independent experiments. #, *P *value of < 0.01 compared to bacteria-stimulated PCPECs, * *P *value of < 0.05 compared to *S. suis *strain 10.

As previously described, the mRNA expression for ICAM-1 and VCAM-1 is augmented in the PCPEC standard Transwell system under TNFα stimulation [26]. We now analysed mRNA expression of these cell adhesion molecules (CAMs) under bacterial infections with the encapsulated *S. suis *strain 10 and its non-encapsulated isogenic mutant strain 10 Δcps in comparison to unstimulated control and TNFα-stimulated cells in the inverted Transwell system (Figure 13). Using a quantitative real time-PCR technique we could observe that the mRNA expression levels for ICAM-1 and VCAM-1 remained low after 2 h of bacterial stimulation but already increased significantly under TNFα stimulation. The upregulation of VCAM-1 (-ΔΔC_T _7.5 ± 1.1) was more intensive than that of ICAM-1 (-ΔΔC_T _3.02 ± 0.08). After 4 h of stimulation, an intense upregulation of ICAM-1 as well as VCAM-1 could be detected after stimulation with *S. suis*, too. Upregulation of VCAM-1 was again significantly stronger than that of ICAM-1 for all stimuli. For both CAMs the mRNA expression induced by the non-encapulated mutant was higher than by the wild-type *S. suis *strain after 4 hours stimulation time but this was significant only for ICAM (ICAM-1: Strain 10 -ΔΔC_T _1.5 ± 0.1, strain 10 Δcps -ΔΔC_T _2.6 ± 0.2; VCAM-1: Strain 10 -ΔΔC_T _4.5 ± 1.7, strain 10 Δcps -ΔΔC_T _6.4 ± 1.2). TNFα caused the strongest effect on CAM expressions in PCPECs and was significantly higher compared to bacteria-stimulated cells (ICAM-1: -ΔΔC_T _5.5 ± 0.5, VCAM-1: -ΔΔC_T _10.8 ± 1.1).

## Discussion

One crucial step in the pathogenesis of bacterial meningitis after bacterial invasion into the CNS is the excessive infiltration of leukocytes into the cerebrospinal fluid leading to massive inflammation. To study PMN transmigration at the blood-CSF barrier, we used a recently established inverted Transwell filter model of PCPECs that enables investigation of leukocyte transmigration in the physiologically relevant basolateral-to-apical direction. [4]. This model displayed robust barrier properties, which were not compromised by PMNs alone or the chemoatractant IL-8. The chemokine IL-8 (CXCL8) is known to interact with its cognate receptors CXCR1 and CXCR2. CXCR2 is the main receptor involved in neutrophil chemotaxis, leading to cell migration into the brain during injury, infection or disease [30]. Interestingly, although bacterial infection alone did not influence barrier function, TEER and paracellular TR-dextran flux were significantly affected during the process of PMN traversal under bacterial stimulation, an effect that was already observed after treatment with TNFα alone. Nevertheless, the increased TR-dextran flux alone might not lead to paracellular transmigration of PMNs, since the observed compromise of tight junction function is still much lower than that of our own previously published results in the standard Transwell system after *S. suis *infection [3,11]. Conflicting data exist as to whether PMN transmigration itself can lead to significant barrier disruption, and this seems to depend on the cells and the stimulus used [27,31-33].

The alteration of barrier function after PMN transmigration following infection with *S. suis *or by TNFα stimulation was associated with an altered actin cytoskeleton and TJ morphology. Independently of the presence of PMNs, both stimulations led to stress fiber formation and an actin rearrangement within the cells, and a fuzzy and blurred distribution pattern of ZO-1 and occludin. In contrast, TEER of PCPECs was not significantly affected by *S. suis *infection alone. Corresponding changes in PCPECs in immunofluorescence analyses of tight junction and cytoskeletal actin morphology were not as strong as described earlier by our group and Zeni and co-workers in the standard Transwell system, which might explain the lower effect on barrier function [3,26]. Interestingly, in the present study the TJ structure was specifically altered in areas where PMNs crossed the PCPEC monolayer between cells (as described in Figures 5, 6, 7, 8). Therefore, local changes in PCPEC morphology may correspond to minor or no TEER decrease. Changes in actin rearrangement may also not necessarily go along with a dramatic decrease of TEER. To maintain barrier function during paracellular transepithelial migration, close cell-cell contact and highly regulated mechanisms are necessary for opening and closing of the TJ [34,35].

Infection with the well-encapsulated *S. suis *wild-type strain 10 and stimulation with the proinflammatory cytokine TNFα led to an augmented PMN traversal in both the presence and absence of IL-8. This goes in line with results that TNFα stimulation of HBMEC or renal proximal tubular cells leads to increased PMN transmigration [27,36]. Interestingly, the stimulation of PCPECs with *S. suis *strain 10 Δcps caused no increase of PMN transmigration. *S. suis *strain 10 Δcps may have induced cell signalling in PCPECs or PMNs that prevented PMN transmigration. Previous results of Chabot-Roy et al. have shown an increased PMN phagocytosis rate of *S. suis *strain 10 Δcps compared to the wild-type, which may also influence the transmigration capacity of PMN [37]. Further experiments are necessary to clarify these questions.

For transendothelial migration a paracellular route between adjacent cells has been postulated for a long time, but in the meanwhile the transcellular route directly through the endothelial cell body has been well documented [14,15]. In contrast, there is to date no evidence that PMNs take the transcellular route through epithelial cells [12,13]. The concert of experimental techniques applied in this study consistently suggests a general preference of neutrophils for transcellular transmigration pathways across PCPECs. By means of immunofluorescence and transmission and scanning electron microscopy analyzes we were indeed able to provide evidence for migratory events through one single cell. However, in determining the transmigration route of PMNs through PCPECs, we were confronted with the problem that neither our immunofluorescence nor electron microscopic images were absolutely unequivocal. If, for example, a leukocyte appears to lie inside a plexus epithelial cell, this could be interpreted simply as transcellular migration. However, the cell migrating along the intercellular cleft could be sectioned in a way that would allow us to interpret the position of the cell falsely as an intracellular one. Intracellular location in this context is defined as a PMN that is in a vacuolar structure and not in the cytoplasm. By definition, guest cell and host cell form a double membrane. However, the difficulty consists in determining whether the guest cell is only surrounded by the host cell in the section plane, while outside the section plane the cell could have contact with the extracellular space resembling an incomplete phagocytosis. We therefore compared orthogradely sectioned filters with filters sectioned nearly parallel through the apical surface to allow better interpretation of cellular details during transmigration. In every case the maintenance of the tight junctions was definitely observed by electron microscopy. Whether or not tight junctions in this system can be opened cannot be answered for principal reasons: we have currently no means to identify an opened junction. However, both *S. suis *or TNFα stimulation led to a fuzzy and blurred distribution pattern of ZO-1 as well as stress fiber formation and actin rearrangement within the cells as described earlier by our group and Zeni and co-workers in the standard Transwell system that may still promote paracellular PMN transmigration [3,26]. In scanning electron microscopic images PMNs were regularly found directly on the apical surface of the monolayer and at a distance from the intercellular clefts. Our data are in line with very recent findings by von Wedel-Parlow et al. [38], who demonstrated, in an in vitro model of the blood-brain barrier under inflammatory conditions, that PMNs preferentially migrate across primary cultured porcine brain capillary endothelial cells via the transcellular route.

Another striking observation in our experiments is that funnel-like structures were regularly found adjacent to PMNs protruding from the apical PCPEC membrane. We observed these funnel-like structures in stimulated as well as unstimulated PCPECs only in the presence of PMNs. The present funnel-like structures are apparently used by transmigratory PMNs, independent of their stimulatory status. However, we could not confirm the possibility that funnel-like structures are a *conditio sine qua non *for the transmigration of PMNs. Although we cannot unequovically exclude a completely paracellular process, our data imply that tight junctions induce a stop of paracellular migration and lead to a deviation of the PMN travelling route to involve a transcellular step via the indented apical compartment of the epithelial cell. This mechanism is to some extent different from the suggested paracellular transmigration of PMNs across the CP after brain injury [39]. In a mouse model it has been described that neutrophils of wild-type mice migrate predominantly paracellularly whereas CD11b/CD18-deficient mice use predominantly the transcellular pathway [40]. The reason for this difference is related to PMNs crawling to cell junctions that are absent in CD11b/CD18-deficient PMNs. The increase in permeability was nearly identical for both forms of migration, because the endothelium forms a dome over the migrating neutrophil, which sealed the emerged gap and thereby limited alterations in vascular permeability independent of the migration route [18]. It should be noted that the two ways of transmigration - paracellular and transcellular - are not mutually exclusive.

The exact routes and molecular mechanisms of PMN transmigration through epithelium are still not fully understood. For T84 intestinal epithelial cells important roles of CD11b/CD18, but also CD11b/CD18-independent mechanisms, have been described under specific inflammatory conditions [31,41]. Along these lines we observed that PMN transmigration through PCPECs is also CD11b/CD18-dependent. Interestingly, we found no evidence for a role of the integrin-associated glycoprotein CD47 during PMN migration through choroid plexus epithelium. In contrast, in intestinal epithelial cells CD47 plays an essential role in PMN transepithelial migration and in signalling events involving the signal regulatory protein α (SIRPα) [12]. Other potential binding partners of PMNs on the surfaces of PCPECs are ICAM-1 for the β_2_-integrins and VCAM-1 for the β_1_-integrins. In cultured intestinal epithelial monolayers ICAM-1 is not involved in PMN transmigration and is not a counter receptor for CD11b/CD18 on the basolateral cell side of the cells [42]. Instead, CD11b/CD18 promotes adherence of PMNs at the apical cell surface and, rather, binding to fucosylated glycoproteins at the basolateral cell side [43,44]. In contrast, the transendothelial migration of most leukocytes has been shown to be ICAM-1-, VCAM-1-, CD11a/CD18- and CD11b/CD18-dependent [15]. Ultrastructural studies in an experimental autoimmune encephalomyelitis model revealed polar localization of ICAM-1, VCAM-1, and MAdCAM-1 on the apical surface of choroid plexus epithelial cells and their complete absence on the fenestrated endothelial cells within the choroid plexus parenchyma [6].

Since contrary data exist about the role of ICAM-1 and VCAM-1 in PMN adhesion and transmigration as described above we were interested in their role on PCPECs after infection with *S. suis*. We analyzed mRNA levels for these adhesion molecules in PCPECs under bacterial infection with *S. suis *strain 10 and its isogenic mutant strain 10 Δcps and after TNFα stimulation, using quantitative real time-PCR. The mRNA expressions of ICAM-1 and VCAM-1 were considerably upregulated with VCAM-1 to a greater extent than ICAM-1 and with TNFα as the strongest stimulus. ICAM-1 upregulation was significantly more enhanced after infection with *S. suis *strain 10 Δcps. Thus, we speculate that this effect may be due to the higher invasion capacity of this strain as recently demonstrated [4]. These data indicate a certain involvement of the adhesion molecules on PCPECs after *S. suis *infection. Further studies are necessary to determine the exact localization of these CAMs on PCPECs and their binding partners.

## Conclusion

The present study gives new insights in PMN transepithelial migration and describes for the first time the transcellular pathway of PMNs through epithelial cells as the final step in diapedesis. Moreover, we were able to demonstrate mechanisms of PMN transmigration through the blood-CSF barrier after *S. suis *infection and TNFα stimulation *in vitro*, which could be relevant for therapeutic strategies in controlling neutrophilic inflammation.

## Competing interests

The authors declare that they have no competing interests.

## Authors' contributions

TT and CW conceived and coordinated the study, and drafted the manuscript. CW, AS, UQ performed transmigration, immunofluorescence, qReal Time-PCR and FACS experiments. HW, LG, MAS, JB, UQ performed the electron microscopic studies. HJG, CS, HS have co-conceived the study and have been involved in drafting the manuscript. All authors have read and approved the final version of this manuscript.
